# EVIDENCE SUPPORTING CERAMIDE ACCUMULATION AS A NOVEL HALLMARK OF AGING

**DOI:** 10.1093/geroni/igae098.2253

**Published:** 2024-12-31

**Authors:** Laila Scroggins, Klarisse Echevarria, Eduardo Gutierrez, Juan Palavicini, Penelope Quiles

**Affiliations:** Howard University, Chesapeake, Virginia, United States; University of Texas Health Science Center at San Antonio, San Antonio, Texas, United States; The Barshop Institute of Longevity and Aging Studies, San Antonio, Texas, United States; UT Health SA, San Antonio, Texas, United States; The Barshop institute of Longevity and Aging, San Antonio, Texas, United States

## Abstract

Aging is the single greatest risk factor for all major chronic diseases, and thus, understanding the biological mechanisms that modulate aging is critical for the development of health-maximizing interventions. Lipids are small hydrophobic molecules that not only serve as fundamental cellular components, but also act as important cellular signaling molecules, with well-established roles in nutrition, health, and disease. The first longevity gene discovered in yeast, i.e. the longevity-assurance gene (LAG1), encodes an enzyme that synthesizes ceramides, a lipid class that has been associated with insulin resistance, apoptosis, mitochondrial dysfunction, senescence, inflammation, sarcopenia, frailty, among other age-related pathways and phenotypes. Using a number of animal models and methodologies, we have amassed a significant amount of data that place ceramides as a novel major driver of aging. Specifically, we found that: (1) ceramides consistently accumulate with age in circulation and in multiple organs in multiple mouse strains and marmosets; (2) circulating ceramides are dramatically reduced in long-lived isolated growth hormone deficient mice; (3) hepatic ceramides are substantially reduced in old marmosets treated with rapamycin, a well-established anti-aging drug, through a mechanisms that seems to involve an acid ceramidase (ASAH1); (4) low doses of myriocin, a potent inhibitor of ceramide synthesis, improves healthspan (i.e. glucose homeostasis and grip strength) in WT C57BL/6J, Balb/c, and UM-HET3 mice fed with Western diet. In addition, preliminary data suggests that myriocin significantly extends lifespan in mice as well. Taken together, our results place ceramide accumulation as a novel driver of aging.

